# Checklist and phenetics studies of nymphs of two species of triatomines: *Triatoma lenti* Sherlock & Serafim, 1967 and *Triatoma sherlocki* Papa, Jurberg, Carcavallo, Cerqueira, Barata, 2002 (Hemiptera: Reduviidae: Triatominae)

**DOI:** 10.1590/0037-8682-0394-2021

**Published:** 2021-12-17

**Authors:** Leandro Augusto Rosseto, Vinícius Fernandes De Paiva, Tiago Belintani, Jader de Oliveira, Vagner José Mendonça, João Aristeu Da Rosa

**Affiliations:** 1 Universidade Estadual de São Paulo, Faculdade de Ciências Farmacêuticas, Araraquara, SP, Brasil.; 2 Universidade Estadual de Campinas, Instituto de Biologia, São Paulo, SP, Brasil.; 3 Universidade de São Paulo, Departamento de Epidemiologia, São Paulo, SP, Brasil.; 4 Universidade Federal do Piauí, Departamento de Parasitologia e Microbiologia, Teresina, PI, Brasil.

**Keywords:** Chagas disease, Morphometric, Morphology of nymphal, Scanning electron microscope, Taxonomy, *Triatoma* immature instars

## Abstract

**INTRODUCTION:**

*Triatoma lenti* and *Triatoma sherlocki* are endemic species of the State of Bahia, located in northeastern Brazil, where they have records of domiciliation in the human environment. In view of the epidemiological aspect and taxonomic importance of these species for the systematics of the *Triatoma* genus, a study was carried out with nymphs of all five instars.

**METHODS:**

An extensive review of studies on nymphs from the subfamily Triatominae is presented. Morphology was studied using a scanning electron microscope and morphometric analyses.

**RESULTS:**

The morphological study allowed us to characterize and discriminate species by means of scanning electron microscope of the last abdominal segment. In addition, the results show morphometric variability, with the total size of the head that best discriminates the species.

**CONCLUSIONS:**

Studies on nymphs are fundamental to the ecosystem; however, the literature on the immature forms of certain groups is scarce, difficult to use, or nonexistent. Therefore, this study includes morphological and morphometric data of the nymphal instars of *T. lenti* and *T. sherlocki*, corroborating the specific taxonomy of these species.

## INTRODUCTION

In the Americas, various species of triatomines are vectors of *Trypanosoma cruzi* (Chagas, 1909) (Kinetoplastida, Trypanosomatidae), the etiological agent of Chagas disease^1^. The insects of the subfamily Triatominae (Jeannel, 1919) are hematophagous and feed primarily on vertebrate blood^2^. Even though all species of triatomines are hematophagous, species that colonize residential places or are peridomicile have increased chances of transmitting *T. cruzi* to humans^3^. After the successful *Triatoma infestans* (Klug, 1834) control program conducted by the Brazilian National Health Foundation, other triatomines previously considered predominately sylvatic have emerged as potential vectors in several areas of Brazil^4^ ^,^ ^5^.

Presently, 157 species (including 3 fossils) within 18 genera are recognized as valid in this subfamily^6^ ^,^ ^7^ ^,^ ^8^. The genus with the greatest number of species described is *Triatoma* Laporte, 1832, which includes members of the *Triatoma brasiliensis* subcomplex, *Triatoma brasiliensis* Neiva 1911, *Triatoma brasiliensis macromelasoma* Galvão 1956, *Triatoma melanica* Neiva & Lent, 1941, *Triatoma juazeirensis* Costa & Felix 2007*, Triatoma sherlocki* Papa, Jurberg, Carcavallo, Cerqueira, Barata, 2002*, Triatoma lenti* Sherlock & Serafim, 1967, *T. bahiensis* Sherlock & Serafim, 1967 and *Triatoma petrochiae* Pinto & Barreto 1925.^10^ ^,^ ^11^

Sherlock and Serafim^12^ described *T. lenti*, *T. pessoai*, and *T. bahiensis*. The authors reported that *T. lenti* and *T. pessoai* were naturally infected by *T. cruzi* and were relatively easily maintained in the laboratory by feeding on pigeons. Currently, only *T. pessoai* is not considered a valid species^13^ ^,^ ^14^.

Cerqueira et al.^15^ refer to the encounter of wild triatomine, naturally infected by *T. cruzi* in the district of Santo Inácio, municipality of Gentio do Ouro, Bahia. Later in 1982, Cerqueira, in his doctoral dissertation, studied the biological cycle and evaluated the results of crosses of this wild triatomine with *T. brasiliensis*; however, it was not considered a new species. Papa et al.^16^ resumed studies of the triatomines studied by Cerqueira in 1982 and based on consistent morphological characters, such as genital structures, shorter wings, red orange spots on the connexivum and legs, inability to fly, and longer legs, concluded that it is a new species named *T. sherlocki*. *Triatoma sherlocki* was related to *T. lenti* by morphological characteristics, cytogenetics, molecular data, and experimental crosses, and was included as a member of the *Triatoma brasiliensis* complex^5^ ^,^ ^9^ ^,^ ^10^ ^,^ ^11^ ^,^ ^14^ ^,^ ^16^ ^,^ ^74^.

Morphology and morphometry are tools that contribute to the knowledge of triatomines and generate useful information to establish more effective strategies for vector control^17^. In Triatominae, biometric studies are used to characterize new species, detect populations, and define structures^18^. For example, geometric morphometry allows the collection of information about the shape and size of organisms, which helps in systematic and phylogenetic studies^10^ ^,^ ^19^ ^,^ ^20^. 

Several authors have used morphology and morphometry to characterize the species and correlate the known characteristics of the character, isoenzymatic and ecological, and contributed to both systematic analyses. Studies on immature instars of *T. lenti* and *T. sherlock* are scarce; therefore, we evaluated and characterized those species that make up the *T. brasiliensis* subcomplex, a relevant group for the ecoepidemiology of Chagas disease in the northeastern region of Brazil^21^ ^-^ ^25^, by gathering all information from the literature on the study of immature forms in Triatominae.

## METHODS

### Insects

**FIGURE 1:**
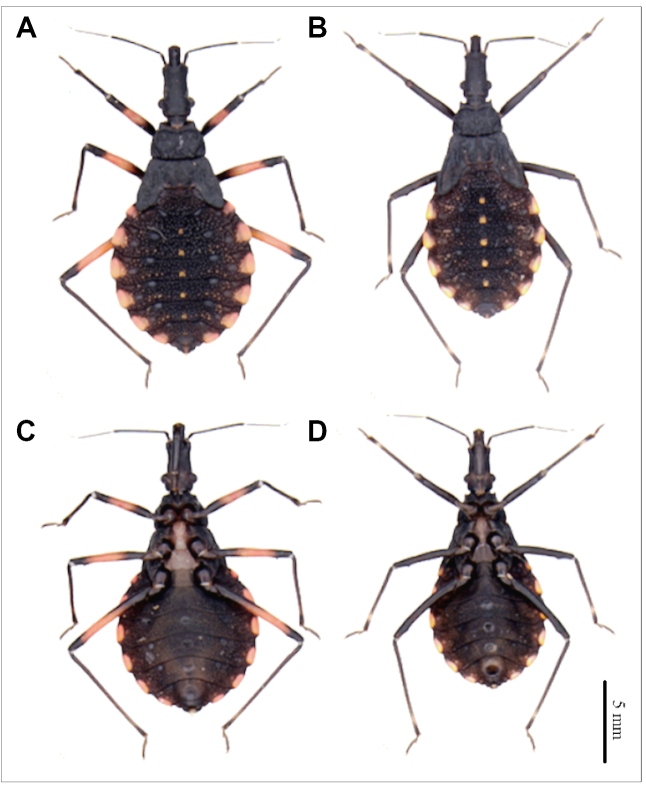
Fifth instar nymph of *T. sherlocki* and *T. lenti.* **(A)** Dorsal view of the fifth instar nymph of *T. sherlocki.* **(B)** Dorsal view of the fifth instar nymph of *T. lenti,* **(C)** Ventral view of the fifth instar nymph of *T. sherlocki*, and **(D)** Ventral view of the fifth instar nymph of *T. lenti.*

We used specimens from a *T. lenti* **(** Figure 1 **)** colony collected on April 9, 2008, which were found in the county of Macaúbas (Mangabeiras and Cana Brava neighborhoods) in the state of Bahia. The specimens were collected at altitudes of 747, 755, 780, and 829 m in the peridomicile and intradomicile. On July 22, 2003, *T. sherlocki* **(** Figure 1 **)** was collected in Gentio do Outo, Santo Inácio, Bahia state, and later a colony was established in the laboratory. The specimens were kept and deposited at the Triatomine Insectario of the Faculty of Pharmaceutical Sciences, Universidade Estadual Paulista (https://www2.fcfar.unesp.br/#!/triatominae). Approved by the Ethics Committee on the Use of Animals - CEUA, CEUA/FCF/CAr: 15/2017).

### Morphological analyses

The fifth instar nymphs from *T. sherlocki* and *T. lenti* **(** Figure 1 **)** were cleaned using an ultrasound device. Next, the structures were dehydrated in alcohol, dried in an incubator at 45ºC for 20 min, and fixed in small aluminum cylinders with transparent glass. Sputtering metallization was then performed on the samples for 2 min at 10 mA in an Edwards sputter coater. After metallization, the samples were studied and photographed using a Topcon SM-300 scanning electron microscope (SEM), according to Rosa et al.^26^. The images obtained were processed (background, contrast, brightness) using the GNU Image Manipulation Program v2.0.2 (GIMP) software free and open-source image editor, and the structures were described and compared.

### Morphometric analyses

For the *T. lenti* and *T. sherlocki* measurements, 15 nymphs specimens in the first, second, third, fourth, and fifth instars were fixed on glass slides using a double-sided tape. Measurements were also taken to determine the thorax, abdomen, and head length, as well as interocular, ante-ocular, and postocular distance, eye diameter, and the three proboscis segments. These distances were defined by Dujardin et al.^27^. The measurements were taken using a Leica MZ APO stereomicroscope and analyzed using the Motic Advanced 3.2 image analysis software. Descriptive statistics analyses and Welch's *t*-test were performed using GraphPad Prism v.5.03. 

### Principal component analysis

To visualize the general patterns of morphological variation in the multidimensional data obtained with the principal component analysis (PCA) of the references, a factorial map was generated using Past 3.2^28^.

### Checklist of studies on the immature instars of triatomines

The survey of publications that study the immature instars of development had as selection criteria publications with morphology and morphometry of nymphs regardless of the methodological approach. Publications were retrieved from databases such as: National Center for Biotechnology Information- NCBI (available at https://www.ncbi.nlm.nih.gov/), Bibliography of Triatominos - BibTri (available at: https://bibtri.cepave.edu. ar/webbibtri.php), Google Academic (https://scholar.google.com.br/?hl=pt) and Scielo (https://www.scielo.org/). The keywords used for the search were: Nymphs, Triatominae, Hemiptera, Reduviidae, Morphology, Morphometry, Description, Ontogenetic, Instar, Description of nymphs, key, eggs, 1st, 2nd, 3rd, 4th, 5th and instars.

## RESULTS

Through an extensive literature survey on immature forms of triatomines, we recovered 115 studies that explored the morphological aspects of nymphs; therefore, we updated the list presented by Galvão 2014^6^ (Table 1).

**TABLE 1:** Checklist of studies that include information on the nymphal instars of triatomines (Galvão, 2014^6^, with modifications).

Species	Approach	References
*Alberprosenia goyavargasi*	Description of nymphs by SEM*	Carcavallo et al.^29^
*Alberprosenia malheiroi*	Description of nymphs of 1^st^, 2^nd^, 3^rd^, 4^th^, and 5^th^ instars (MO*, SEM)	Carcavallo et al.^30^
*Belminus herreri*	Description and geometric morphometry of nymphs	Rocha et al.^31^
*Cavernicola pilosa*	Description of nymphs of 1^st^, 2^nd^, 3^rd^, 4^th^, and 5^th^ instars (MO)	Valderrama & Lizano^32^
*Cavernicola lenti*	Description of nymphs (MO), every aspect shown by SEM	Costa et al.^33^
*Dipetalogaster maxima*	Description of nymphs of 1^st^, 2^nd^, 3^rd^, 4^th^, and 5^th^ instars (MO)	Jurberg et al.^34^
*Eratyrus mucronatus*	Morphological (MO and SEM) and key	Galíndez-Girón et al.^35^
*Linshcosteus confumus*	SEMs and description of eggs	Haridass^36^
*Linshcosteus costalis*	SEMs and description of eggs	Haridass^36^
*Linshcosteus karupus*	Description of nymphs of 1^st^, 2^nd^, 3^rd^, 4^th^, and 5^th^ instars by SEM	Galvão et al.^37^
*Triatoma longipennis*	Morphological (MO and SEM) and key	Galíndez-Girón et al.^35^
*Triatoma pallidipennis*	Ontogenetic morphometrics (MO)	Rodríguez-Sánchez et al.^38^
	Morphological (MO and SEM) and key	Galíndez-Girón et al.^35^
*Triatoma phyllosoma*	Morphological (MO and SEM) and key	Galíndez-Girón et al.^35^
*Mepraia spinolai*	Morphological (MO and SEM) and key	Galíndez-Girón et al.^35^
*Microtriatoma trinidadensis*	Description of nymphs (MO)	Carcavallo et al.^39^
		Riva et al.^40^
*Nesotriatoma flavida*	Morphometrics	Jiménez and Fuentes,^41^
*Panstrongylus geniculatus*	Morphological and key (MO and SEM)	Galíndez-Girón et al.^35^
*Panstrongylus humeralis*	Morphological and key (MO and SEM)	Galíndez-Girón et al.^35^
*Panstrongylus lignarius*	Morphological and key (MO and SEM)	Galíndez-Girón et al.^35^
*Panstrongylus megistus*	Morphology of spiracles 5^th^ instar nymphs	Rosa et al.^26^
	Sexual distinction between 5^th^ instar nymphs by SEM	Rosa et al.^42^
	Morphology of 5^th^ instar nymphs by SEM	Rosa et al.^43^
	Abdominal structures of 5^th^ instar nymphs	Rosa & Barata^44^
	Morphology of antennae of 1^st^, 2^nd^, 3^rd^, 4^th^, and 5^th^ instars (SEM)	Rosa et al.^45^
*Paratriatoma hirsuta hirsuta*	Morphology of 5^th^ instar nymphs (MO)	Ryckman^46^
	Morphological study (MO and SEM)	Galíndez-Girón et al.^35^
*Paratriatoma hirsuta kamiensis*	Morphology of 5^th^ instar nymphs (MO)	Ryckman^46^
*Paratriatoma hirsuta papagoensis*	Morphology of 5^th^ instar nymphs (MO)	Ryckman^46^
*Paratriatoma hirsuta pimae*	Morphology of 5^th^ instar nymphs (MO)	Ryckman^46^
*Paratriatoma hirsuta yumanensis*	Morphology of 5^th^ instar nymphs (MO)	Ryckman^46^
*Paratriatoma lecticularia*	Description of nymphs (MO) and visualization of structures using SEM.	Rocha et al.^47^
	Morphological and key (MO and SEM)	Galíndez-Girón et al.^35^
*Psamolestes arthuri*	Description of nymphs of 1^st^, 2^nd^, 3^rd^, 4^th^, and 5^th^ instars (MO)	Carcavallo et al.^48^
*Psamolestes coreodes*	Morphological (MO and SEM) and key	Galíndez-Girón et al.^35^
*Rhodnius brethesi*	Description of nymphs of 1^st^, 2^nd^, 3^rd^, 4^th^, and 5^th^ instars	Mascarenhas^50^
*Rhodnius dalessandroi*	Morphological and key (MO and SEM)	Galíndez-Girón et al.^35^
*Rhodnius ecuadoriensis*	Morphological and key (MO and SEM)	Galíndez-Girón et al.^35^
*Rhodnius neglectus*	Morphology of spiracles 5^th^ instar nymphs	Rosa et al.^26^
	Sexual distinction between 5^th^ instar nymphs by SEM	Rosa et al.^42^
	Morphology of 5^th^ instar nymphs by SEM	Rosa et al.^43^
	Abdominal structures of 5^th^ instar nymphs	Rosa & Barata,^44^
	Morphometric of 1^st^, 2^nd^, 3^rd^, 4^th^, and 5^th^ instars (MO)	Ponsoni et al.^49^
	Morphology of antennae of 1^st^, 2^nd^, 3^rd^, 4^th^, and 5^th^ instars (SEM)	Rosa et al.^45^
*Rhodnius neivai*	Morphological and key (MO and SEM)	Galíndez-Girón et al.^35^
*Rhodnius pallescens*	Morphological and key (MO and SEM)	Galíndez-Girón et al.^35^
*Rhodnius prolixus*	Morphometric of 1^st^, 2^nd^, 3^rd^, 4^th^, and 5^th^ instars (MO)	Marconato et al.^51^
	Morphology of antennae of 1^st^, 2^nd^, 3^rd^, 4^th^, and 5^th^ instars (SEM)	Rosa et al.^45^
		Rosa et al.^26^
	Morphology of spiracles 5^th^ instar nymphs	Rosa et al.^42^
	Sexual distinction between 5^th^ instar nymphs by SEM	Rosa et al.^43^
	Morphology of 5^th^ instar nymphs by SEM	Rosa & Barata,^44^
	Abdominal structures of 5^th^ instar nymphs	Ponsoni et al.^49^
	Morphology and key (MO and SEM)	Galíndez-Girón et al.^35^
*Rhodnius pictipes*	Description of nymphs by MO	Lent & Valderrama^52^
*Triatoma arthurneivai*	Nymphal instars by SEM	Rosa et al. ^87^
*Triatoma baratai*	Description of nymphs (MO) and visualization of structures using SEM	Rocha et al.^53^
*Triatoma barberi*	Description of nymphs of 1^st^, 2^nd^, 3^rd^, 4^th^, and 5^th^ instars (MO, SEM)	Carcavallo et al.^30^
*Triatoma brasiliensis*	Description of nymphs (MO) and visualization of structures using SEM	Jurberg et al.^54^
*Triatoma breyeri*	Description and keys for all instars.	Rosa & Barata,^44^
	Morphological and key (MO and SEM)	Galíndez-Girón et al.^35^
*Triatoma carcavalloi*	Description of nymphs of 1^st^, 2^nd^, 3^rd^, 4^th^, and 5^th^ instars (MO)	Jurberg et al.^55^
*Triatoma circummaculata*	Morphology of the head of 1^st^ and 5^th^ instar nymphs and visualization of some structures by SEM	Rosa et al.^55^ ^,^ ^56^
*Triatoma costalimai*	Description and ontogenetic morphometrics of instars	Raigorodschi et al^58^
*Triatoma deaneorum*	Description of nymphs of 1^st^, 2^nd^, 3^rd^, 4^th^, and 5^th^ instars (MO)	Galvão &Fuentes ^59^
*Triatoma delpontei*	Morphological and key (MO and SEM)	Galíndez-Girón et al.^35^
	Description and keys for all instars	Brewer et al.^60^ ^,^ ^61^
*Triatoma dimidiata*	Morphological study of nymphs (MO and SEM)	Mello et al.^62^
*Triatoma dispar*	Morphological and key (MO and SEM)	Galíndez-Girón et al.^35^
*Triatoma eratyrusiformis*	Morphological and key (MO and SEM)	Galíndez-Girón et al.^35^
*Triatoma gerstaeckeri*	Morphological study of nymphs (MO and SEM)	Galíndez-Girón et al.^35^
*Triatoma guasayana*	Description of nymphs of 1^st^, 2^nd^, 3^rd^, 4^th^, and 5^th^ instars (MO)	Brewer & Garay^63^
*Triatoma guazu*	Description of nymphs of 1^st^, 2^nd^, 3^rd^, 4^th^, and 5^th^ instars (MO and SEM)	Silva et al.^64^
	Comparative study of stridulatorium sulcus, bucculla and rostrum	Silva et al.^65^ ^,^ ^83^
	Morphological and key (MO and SEM)	Galíndez-Girón et al.^35^
*Triatoma infestans*	Sexual distinction between 5^th^ instar nymphs by SEM	Rosa et al.^26^
	Morphology of 5^th^ instar nymphs by SEM	Rosa et al.^42^
	Abdominal structures of 5^th^ stage nymphs	Rosa et al.^43^
	Description of nymphs of 1^st^, 2^nd^, 3^rd^, 4^th^, and 5^th^ instars (MO)	Galíndez-Girón et al.^35^
*Triatoma jurbergi*	Description of nymphs of 1^st^, 2^nd^, 3^rd^, 4^th^, and 5^th^ instars (MO, MEV)	Jurberg et al.^66^
	Comparative study of stridulatorium sulcus, bucculla and rostrum	Silva et al.^67^
*Triatoma klugi*	Description of nymphs (MO) and visualization of structures using SEM.	Jurberg et al.^66^ ^,^ ^85^
	Comparative study of the stridulatorium sulcus, buccula and rostrum of nymphs	Silva et al.^67^
*Triatoma lenti*	Morphological and key (MO and SEM)	Galíndez-Girón et al.^35^
*Triatoma maculata*	Morphometric of 1^st^, 2^nd^, 3^rd^, 4^th^, and 5^th^ instars (MO)	Gonçalves et al.^71^
	Morphological and key (MO and SEM)	Galíndez-Girón et al.^35^
*Triatoma matogrossensis*	Abdominal structures of 5^th^ instar nymphs	Rosa et al.^26^
	Sexual distinction between 5^th^ instar nymphs	Rosa et al.^42^
	Morphology of 5^th^ instar nymphs by SEM	Rosa et al.^43^
*Triatoma melanocephala*	Morphometric characterization of the nymphal instars	Oliveira et al.^68^ Jurberg et al.^84^
*Triatoma melasoma*	Morphological study of nymphs (MO and SEM)	Galíndez-Girón et al.^35^
*Triatoma nitida*	Morphology of of 1^st^, 2^nd^, 3^rd^, 4^th^, and 5^th^ instars (SEM)	Jurberg et al.^69^
*Triatoma pintodiasi*	Description of nymphs of 1^st^, 2^nd^, 3^rd^, 4^th^, and 5^th^ instars (MO)	Motta & Moreira,^70^
*Triatoma platensis*	Description of nymphs (MO) and keys	Brewer et al.^60^
		Brewer & Garay,^63^
*Triatoma protacta*	Morphology and key of 5^th^ instar nymphs of species and subspecies	Ryckman^82^
*Triatoma pseudomaculata*	Morphometric of 1^st^, 2^nd^, 3^rd^, 4^th^, and 5^th^ instar (MO)	Gonçalves et al.^71^
	Morphology of nymphs (MO and SEM)	Galíndez-Girón et al.^35^
*Triatoma ryckmani*	Description of all immature instars based on MO and SEM	Rocha et al.^88^
*Triatoma rubrofasciata*	SEM	Haridass^36^
*Triatoma rubrovaria*	Morphology of the head of 1^st^ and 5^th^ instar nymphs	Rosa et al.^56^
	Antenna morphometry	Rosa et al.^57^
	Morphological and key (MO and SEM)	Galíndez-Girón et al.^35^
*Triatoma sordida*	Morphometry of 1^st^, 2^nd^, 3^rd^, 4^th^, and 5^th^ instars	Brewer et al.^60^
	Description of nymphs of 1^st^, 2^nd^, 3^rd^, 4^th^, and 5^th^ instars (MO)	Brewer et al.^61^
	and keys for all instars	Brewer & Garay.^63^
	Morphology and key (MO and MEV)	Galíndez-Girón et al.^35^
*Triatoma tibiamaculata*	Abdominal structures of 5-instar nymphs	Rosa & Barata,^44^
*Triatoma vandae*	Description of nymphs (MO) and visualization of structures using SEM.	Silva et al.^72^
	Comparative study of the stridulatorium sulcus, buccula and rostrum of nymphs	Silva et al.^67^
*Triatoma vitticeps*	Antenna morphometry and morphology	Rosa et al.^45^
	Morphology compared to other Reduviidae	Weirauch^73^
*Triatoma williami*	Description of nymphs (MO) and visualization of structures using SEM.	Silva et al.^72^
	Comparative study of the stridulatorium sulcus, buccula and rostrum of nymphs	Silva et al.^67^
	Morphology and key (MO and SEM)	Galíndez-Girón et al.^35^
Diverse species	Some structures and key for Triatominae (MO and SEM)	Galíndez-Girón *et al.* ^35^

### Morphological characteristics

**FIGURE 2:**
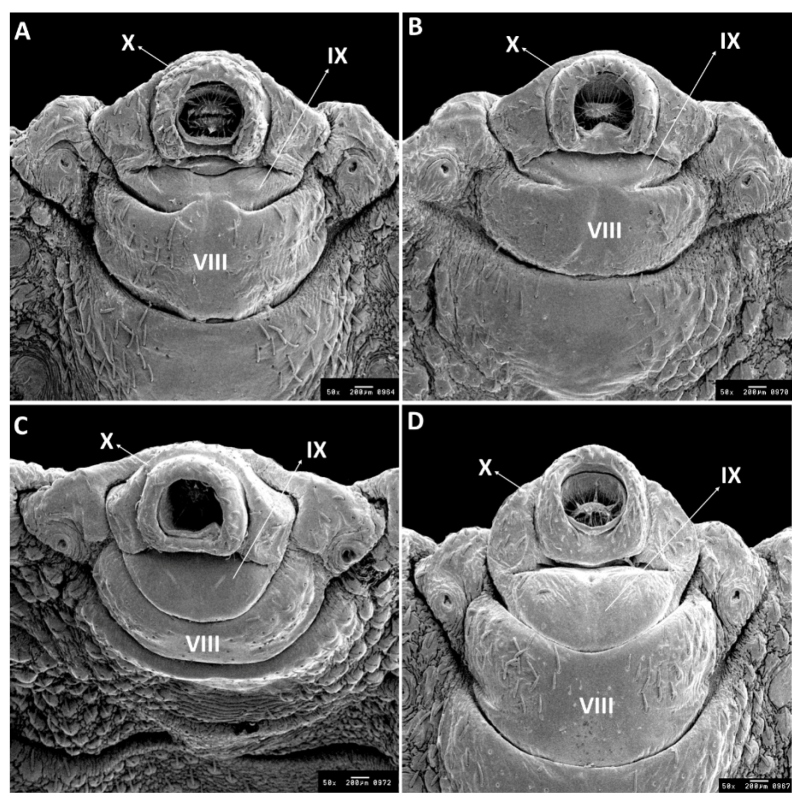
Ventral view of the terminal segments of the male and female fifth instar nymph of *T. sherlock* and *T. lenti*. **(A)** Female of *T. sherlocki*, **(B)** Female of *T. lenti*, **(C)** Male of *T. sherlocki*, **(D)** Male of *T. lenti.* X, IX, VIII: tenth, ninth, eighth ventral abdominal segment.

The morphological characteristics of the two species are presented in Figure 2. According to the genital morphology of fifth instar nymphs, the ninth ventral abdominal segment is wider in *T. lenti* than in *T. sherlocki*, as well as the presence of a hole in the posterior portion of this segment in *T. lenti* and its absence in *T. shelocki*. The ninth ventral abdominal segment of fifth instar nymphs shows parallel grooves in the posterior region, which are most evident in *T. lenti*, while their presence in *T. sherlocki* is poorly visible. The eighth segment was trapezoidal in *T. sherlocki* and oval in *T. lenti.* The laterals were irregular at the apex. It was found that the ninth segment had few sensilla, as well as segments 7, 8, and 10. The tenth segment was curved ventrally in the posterior portion. Sexual dimorphisms of the nymphs are characterized by the size of the ninth segment ventrally, in which females have a narrow (Figure 2 A, B) while males have a wide ninth segment (Figure 2 C, D). 

### Morphometric characteristics

Morphometric characteristics of the two species are presented in Table 2. The averages of the total length of heads in *T. sherlocki* and *T. lenti* were 1.34 and 1.42 mm, 1.84 and 1.83 mm, 2.65 and 2.93 mm, 3.61 and 3.86 mm, and 4.56 and 4.53 mm in the first, second, third, fourth, and fifth instar, respectively. 

**TABLE 2:** Mean and standard deviation results of head (including Outer distance between the eyes, Inner distance between the eyes, Postocular distance, Ante-Ocular distance), thorax and abdomen measurements of *T. lenti* and *T. sherlocki* nymphs.

Parameters	*Triatoma sherlocki*					*Triatoma lenti*				
	1^st^ instar	2^nd^ instar	3^rd^ instar	4^th^ instar	5^th^ instar	1^st^ instar	2^nd^ instar	3^rd^ instar	4^th^ instar	5^th^ instar
Total lenght of head (mm)	1,34 ± 0,03	1,84 ± 0,05	2,65 ± 0,06	3,61 ± 0,17	4,56 ± 0,14	1,42 ± 0,08	1,83 ± 0,05	2,93 ± 0,06	3,86 ± 0,12	4,39 ± 0,16
Outer distance between the eyes (OE) (mm)	0,25 ± 0,01	0,39 ± 0,03	0,53 ± 0,03	0,69 ± 0,04	0,94 ± 0,04	0,24 ± 0,01	0,36 ± 0,03	0,53 ± 0,04	0,70 ± 0,03	0,93 ± 0,03
Inner distance between the eyes (IE) (mm)	0,51 ± 0,01	0,66 ± 0,02	0,92 ± 0,03	1,23 ± 0,07	1,54 ± 0,05	0,55 ± 0,02	0,68 ± 0,02	1,01 ± 0,03	1,29 ± 0,03	1,69 ± 0,07
Postocular distance (PO) (mm)	0,24 ± 0,02	0,31 ± 0,03	0,41 ± 0,02	0,53 ± 0,04	0,60 ± 0,04	0,28 ± 0,03	0,30 ± 0,02	0,48 ± 0,03	0,60 ± 0,04	0,88 ± 0,04
Ante-Ocular distance (AO) (mm)	0,83 ± 0,02	1,20 ± 0,04	1,81 ± 0,04	2,50 ± 0,01	3,21 ± 0,12	0,89 ± 0,04	1,22 ± 0,04	1,95 ± 0,05	2,66 ± 0,07	3,65 ± 0,15
Total thorax lenght (mm)	0,98 ± 0,03	1,43 ± 0,05	2,08 ± 0,06	2,93 ± 0,15	4,68 ± 0,18	0,99 ± 0,05	1,43 ± 0,05	2,02 ± 0,05	3,26 ± 0,11	5,29 ± 0,15
Total abdomen lenght (mm)	1,75 ± 0,11	3,17 ± 0,24	5,24 ± 0,23	6,68 ± 0,42	11,29 ± 0,39	1,57 ± 0,10	3,04 ± 0,13	5,60 ± 0,22	6,33 ± 0,33	11,14 ± 0,75
Proboscis 1^st^ segment (1S) (mm)	0,29 ± 0,03	0,43 ± 0,02	0,58 ± 0,04	0,84 ± 0,04	1,18 ± 0,07	0,29 ± 0,03	0,40 ± 0,02	0,6 ± 0,04	0,85 ± 0,03	1,19 ± 0,06
Proboscis 2^nd^ segment (2S) (mm)	0,65 ± 0,02	1,0 ± 0,04	1,35 ± 0,04	1,85 ± 0,07	2,50 ± 0,08	0,64 ± 0,02	0,98 ± 0,03	1,48 ± 0,03	1,94 ± 0,06	2,58 ± 0,09
Proboscis 3^rd^ segment (3S) (mm)	0,35 ± 0,02	0,54 ± 0,01	0,69± 0,02	0,93 ± 0,03	1,26 ± 0,05	0,36 ± 0,02	0,52 ± 0,02	0,72 ± 0,02	0,97 ± 0,02	1,22 ± 0,06

The averages of the total length of thoraxes in *T. sherlocki* and *T. lenti* were 0.98 and 0.99 mm, 1.43 and 1.43 mm, 2.08 and 2.02 mm, 2.93 and 3.26 mm, and 4.68 and 5.29 mm in the first, second, third, fourth, and fifth instar, respectively. 

The averages of the total length of abdomens in *T. sherlocki* and *T. lenti* were 1.75 and 1.57 mm, 3.17 and 3.04 mm, 5.24 and 5.60 mm, 6.68 and 6.63 mm, and 11.29 and 11.14 mm in the first, second, third, fourth, and fifth instar, respectively. The mean lengths of the abdomen were larger than those of the head, which were larger than those of the thorax in the first stage nymphs in both *T. lenti* and *T. sherlocki*. As in the first stage nymphs, the average abdominal length in second instar nymphs was longer than those of the head and the thorax nymphs for both species. 

In *T. lenti* and *T. sherlocki*, the highest measurement observed was the total length of the abdomen that was longer than the head as well as the thorax. The average lengths of the abdomen were higher than those of the head as well as those of the thorax for *T. lenti* and *T. sherlocki*. Abdomen and eye diameter measurements showed no significant difference between *T. lenti* and *T. sherlocki* (Table 2. p<0.001). Analyzing the fifth instar nymphs of the two species, we found that the abdomen was the largest segment, and unlike the other nymphal instars, the thorax was larger than the head in the fifth stage nymphs of both species. 

The PO, IE, and AO followed an ascending order: first instar > second instar > third instar > fourth instar > fifth instar, for both species (Table 2). Among these parameters, the largest length was the distance before the eye, and the smallest was the diameter of the eyes and the distance between both *T. lenti* and *T. sherlocki*. 

The first, second, and third proboscis segment lengths were in the following order: first segment > third segment > second (Table 2). In fifth stage nymphs, the second and third segments showed significant differences in their length for both species. In both cases, it was observed that the second segment was larger than the third and this was larger than the first for all nymphal instars (Table 2). After measuring and performing statistical analysis on the three segments of the proboscis in first stage nymphs, it was observed that only the second segment showed a significant difference, while the first and third segments did not show significant differences between *T. lenti* and *T. sherlocki* (Table 3). 

**TABLE 3:** Comparative statistical analysis of *T. lenti* and *T. sherlocki* by Welch's t-test for nymph measurements.

*T. lenti x T. sherlocki*		1^st^ instar		2^nd^ instar		3^rd^ instar		4^th^ instar		5^th^ instar	
		p value	significance	p value	significance	p value	significance	p value	significance	p value	significance
Head	Total lenght	0,0057	**	0,6471	NS	<0,0001	***	0,0001	***	0,0087	**
	OE	0,0517	*	0,0025	**	0,6927	NS	0,2272	NS	0,05686	*
	IE	<0,0001	***	0,0187	*	<0,0001	***	0,0099	*	<0,0001	***
	PO	0,0012	**	0,2312	NS	<0,0001	***	0,0002	**	<0,0001	***
	AO	<0,0001	***	0,2621	NS	<0,0001	***	0,0003	**	<0,0001	***
Proboscis	1S	0,7318	NS	0,0003	**	0,1862	NS	0,3192	NS	0,1506	NS
	2S	0,0338	*	0,0066	**	<0,0001	***	0,0003	**	0,0099	*
	3S	0,3836	NS	0,0076	**	0,0006	**	0,0058	*	0,0396	*
Total thorax lenght		0,4811	NS	0,7834	NS	0,0267	*	<0,0001	***	<0,001	**
Total abdômen lenght		<0,0001	***	0,0923	NS	0,0003	**	0,0186	*	0,4947	NS

Outside distance between the eyes (OE); Inner distance between the eyes (IE); Postocular distance (PO); Ante-Ocular distance (AO), and *NS*, not significant.

Comparisons between proboscis segments and head and abdomen lengths of the two species are presented in Table 3. In the second instar nymphs, the three segments of the proboscis revealed measurements that showed significant differences, according to the statistical analysis, for the two species. In the third and fourth instar nymphs, measurements of the second and third proboscis segments showed a significant difference between *T. lenti* and *T. sherlocki*. 

 In the first instar nymphs, statistical analyses revealed significant differences in total head and abdominal length measurements. Thorax measurements were not different between the two species**.** Regarding the measurements of head parameters of the first stage nymphs, the distance between the anterior, postocular, interocular, and eye diameters were significantly different when comparing *T. lenti* and *T. sherlocki*. Statistical analysis showed significant differences only for interocular distance and eye diameter in second instar nymphs. 

Measurements of the thorax, abdomen, ante-ocular distance, postocular distance, and total head length revealed no significant differences between the two species (Table 3)*.* For the third instar nymphs, all measurements except for the eye diameter and first proboscis segment measurements, showed significant differences between both species, (Table 3). For the fourth instar nymphs, all parameters showed statistically significant differences, except for the eye diameter and first segment of the proboscis, as was also observed for the third instar nymphs (Table 3). The measurements of total head length, ante-ocular distance, postocular distance, and interocular and thorax distance of fifth instar nymphs were significantly different between the two species. 

### Principal component analysis

The main components (PCA1 and PCA2) are presented through biplot graphics showing the morphometric variability between *T. lenti* and *T. sherlocki*. The total size of the head was responsible for greater discrimination between the studied specimens. Alternatively, PC1 and PC2 were 99.569% and 0.431% for the first stage nymphs ( Supp. Figure 1), 99.966% and 0.034% for the second stage (Supp. Figure 2), 99.937% and 0.062% for the third stage (Supp. Figure 3), 99.791% and 0.208% for the fourth (Supp. Figure 4), and 99.84 and 0.15% for the fifth (>Supp. Figure 5).

## DISCUSSION

Studies on immature forms of triatomines are relevant to taxonomy and provide important information for the understanding of several biological aspects of these vectors. In this study, a list of works with immature forms were presented and a morphological characterization of five nymphal instars of *T. lenti* and *T. sherlocki*, species that are closely related phylogenetically^10^ ^,^ ^74^, were described.

*Triatoma lenti* and *T. sherlocki* have reproductive compatibility with other members of the species *T. brasiliensis* subcomplex^5^, which are frequently found in dwellings and infected with *T. cruzi*; therefore, they are potential vectors of Chagas disease^3^ ^,^ ^75^. Costa et al*.* ^76^ conducted a comparative morphological analysis of the external genital structures and eggs of *T. brasiliensis* to differentiate chromatic forms*.* Gonçalves et al.^77^ used classic and geometric morphometry as a tool to distinguish *T. jatai* from other species. Mendonça et al.^14^, used morphological, morphometric, molecular, and cytogenetic approaches as well as experimental crosses to revalidate the specific status of *T. bahiensis* and differentiate it from *T. lenti*. Combining morphometric and molecular approaches has provided important clues about *the T. brasiliensis* complex, which includes the species and subspecies *T. lenti, T. petrocchiae, T. b. brasiliensis, T. b. macromelasoma, T. juazeirensis, T. sherlocki, T. melanica*, and *T. bahiensis* ^10^.

In the present study, using SEM images, morphological differences were observed in the ninth ventral abdominal segment of female and male nymphs of the fifth instar. Comparing the morphology of the ninth ventral abdominal segment of male and female nymphs in the fifth instar of the species *T. melanocephala* Neiva & Pinto, 1923*, T. brasiliensis, T. infestans, T. matogrossensis* Leite and Barbosa, 1953, *T. tibiamaculata* (Pinto, 1926), *T. lenti,* and *T. sherlocki,* it can be seen that these seven species differ by this character^26^ ^,^ ^42^ ^,^ ^78^. This indicates that the shape and size of the ninth abdominal segment in fifth instar nymphs may be taxonomically valid. 

The measurements of the head, thorax, and abdomen served to better characterize and distinguish *T. lenti* and *T. sherlocki* across their evolutionary instars, as well as in the comparative analysis of nymphal instars of other species of the *Triatoma* genus. The combination of morphometric and morphological approaches provides important clues about the delimitation of the complex^26^ ^,^ ^76^ ^,^ ^79^. Oliveira et al*.* ^10^ morphometrically analyzed the species of the *T. brasiliensis* complex and showed that the variations in the shape of the head were statistically significant. The wings showed sexual dimorphism in shape, while the heads were not dimorphic as expected. 

In this study, as in all other nymphal instars, we found that the largest measurement among the head measurements was the anocular distance and the smallest was the postocular distance, In the morphometry, all parameters in the first instar, except the average eye diameter and the first and third proboscis segments, were significantly different between *T. lenti* and *T. sherlocki*. Measurements of interocular distance, eye diameter, and the three segments of the proboscis revealed significant differences between the second instar nymphs of *T. lenti* and *T. sherlocki*. The third and fourth instar nymphs showed significant differences in the measurements of the abdomen, head, thorax, ante-ocular, interocular, postocular, and second and third proboscis segments. In the fifth instar, measurements of thorax length, head length, ante-ocular, interocular, postocular, and second and third proboscis segments showed significant differences in taxonomic differentiation between *T. lenti* and *T. sherlocki*. In all nymphal instars, the total length measurement ratio were in the following order: abdomen > head > thorax. In *Triatoma melanocephala* Neiva & Pinto, 1923*,* the nymphal instars presented the following length pattern: in the first instar, thorax > abdomen > head; in the second instar, abdomen > head > thorax; and in the third, fourth, and fifth instars, abdomen > thorax > head^81^.

In all nymphal instars of *T. lenti* and *T. sherlocki,* it was observed that the second segment of the proboscis was larger than the third which was larger than the first segment. In *T. melanocephala* nymphs, it was found that specimens in the first three nymphs presented the same length order (2 > 3 > 1), while those in the fourth and fifth instars, along with the adults, possessed mouthpart segments of the same order (2 > 1 > 3)^78^. The main components (PCA1 and PCA2) illustrated the differences between the studied parameters and showed that the total size of the head is or that it discriminates against *T. lenti* and *T. sherlocki.*

Studies on nymphs are crucial for the systematic development of certain groups. However, the literature on immature forms of certain groups is scarce, difficult to use, or nonexistent^79^. Epidemiological studies and control measures require precise taxonomic determination of *T. brasiliensis* subcomplex^80^ ^,^ ^86^,^89^. Therefore, this study provides morphological and morphometric data on the nymphal instars of *T. lenti* and *T. sherlocki*, corroborating the specific taxonomy of these species.
