# Supplementary material for: Checklist and phenetics studies of nymphs of two species of triatomines: Triatoma lenti Sherlock & Serafim, 1967 and Triatoma sherlocki Papa, Jurberg, Carcavallo, Cerqueira, Barata, 2002 (Hemiptera: Reduviidae: Triatominae)
Source: Rev Soc Bras Med Trop. 2021 Dec 17;54:e0394-2021. doi: 10.1590/0037-8682-0394-2021 (PMC8687502; doi:10.1590/0037-8682-0394-2021)
Supplement: Supplementary file 3 [file 1678-9849-rsbmt-54-e0394-2021-supp3.pdf]

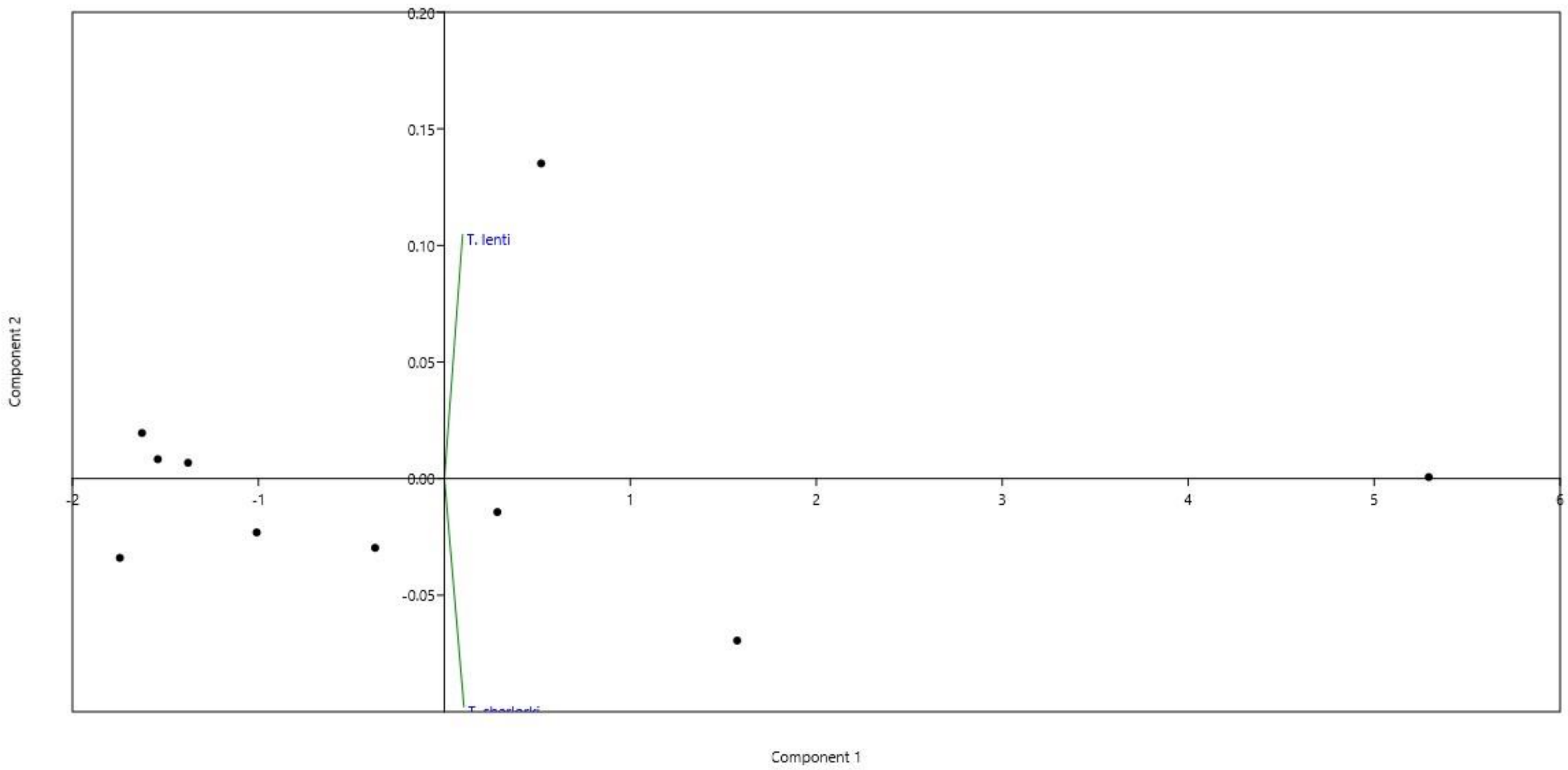

Supp. Figure 3. Biplot for the analysis of the main components of the measurements of nymphs of the 3rd stage of *T. lenti* and *T. sherlocki.*
